# Using computer-vision and machine learning to automate facial coding of positive and negative affect intensity

**DOI:** 10.1371/journal.pone.0211735

**Published:** 2019-02-05

**Authors:** Nathaniel Haines, Matthew W. Southward, Jennifer S. Cheavens, Theodore Beauchaine, Woo-Young Ahn

**Affiliations:** 1 Department of Psychology, The Ohio State University, Columbus, Ohio, United States of America; 2 Department of Psychology, Seoul National University, Seoul, Korea; Universidad Complutense Madrid, SPAIN

## Abstract

Facial expressions are fundamental to interpersonal communication, including social interaction, and allow people of different ages, cultures, and languages to quickly and reliably convey emotional information. Historically, facial expression research has followed from discrete emotion theories, which posit a limited number of distinct affective states that are represented with specific patterns of facial action. Much less work has focused on dimensional features of emotion, particularly positive and negative affect intensity. This is likely, in part, because achieving inter-rater reliability for facial action and affect intensity ratings is painstaking and labor-intensive. We use computer-vision and machine learning (CVML) to identify patterns of facial actions in 4,648 video recordings of 125 human participants, which show strong correspondences to positive and negative affect intensity ratings obtained from highly trained coders. Our results show that CVML can both (1) determine the importance of different facial actions that human coders use to derive positive and negative affective ratings when combined with interpretable machine learning methods, and (2) efficiently automate positive and negative affect intensity coding on large facial expression databases. Further, we show that CVML can be applied to individual human judges to infer which facial actions they use to generate perceptual emotion ratings from facial expressions.

## Introduction

The ability to effectively communicate emotion is essential for adaptive human function. Of all the ways that we communicate emotion, facial expressions are among the most flexible—their universality allows us to rapidly convey information to people of different ages, cultures, and languages. Further, facial expressions signal complex action tendencies including threat and cooperative intent [1–3]. Unsurprisingly, the ability to produce and recognize facial expressions of emotion is of interest to researchers throughout the social and behavioral sciences.

Facial expressions can be interpreted using either message- or sign-based approaches [4]. Message-based approaches describe the meaning conveyed by a facial expression (e.g., happiness), whereas sign-based approaches describe observable facial actions that embody/comprise messages (e.g., cheek raising may indicate happiness). Although message-based approaches are used effectively by psychologists to measure facial expression messages (e.g., happiness), they do not describe facial behavior comprehensively. Instead, they rely on expert judgments of holistic facial expressions—provided by highly trained coders—rather than on facial movements themselves. This renders message-based approaches susceptible to sources of individual differences (unreliability) among human coders that are not inherent to sign-based approaches (e.g., emotional inference on movements after detecting them), which can impede valid comparisons of results across studies and research sites—even when the same construct is measured.

In comparison, multiple comprehensive, standardized sign-based protocols have been developed and used to answer a variety of research questions [4]. Among these protocols, the Facial Action Coding System (FACS; [5]) may be the most widely used. FACS comprises approximately 33 anatomically-based facial actions (termed action units [AUs]), which interact to generate different facial expressions.

Originally developed from a basic emotion theory perspective, the relation between FACS-based AUs and discrete emotions is an active research topic [6]. Distinct patterns of AUs reliably map onto each basic emotion category (happiness, sadness, anger, fear, surprise, and disgust), and the existence of distinct patterns of AUs that people use to label different emotional expressions is often used as evidence to support discrete theories of emotion (see [7]). For example, oblique lip-corner contraction (AU12), together with cheek raising (AU6) reliably signals enjoyment [8], while brow furrowing (AU4) tends to signal negative emotions like anger and sadness (e.g., [9]). Recently, research on how people perceive discrete emotions from AUs has revealed up to 21 discrete categories composed of compound basic emotions (e.g., happily-surprised; [10]). Together, these studies suggest that people use the presence of distinct AUs to evaluate emotional content from facial expressions [11], a hypothesis supported by neuroimaging studies showing that differential patterns of BOLD responding in the posterior superior temporal sulcus discriminate between AUs [12].

Despite the clear links between AUs and discrete emotion perception, little is known about how AUs map onto dimensional features of emotion [7], especially positive and negative affect (i.e., valence). This is a potentially important oversight given the centrality of valance to dimensional theories of emotion (e.g., [13–15]), of which valence is the most consistently replicated dimension [16]. Early work using facial electromyography (EMG) showed that zygomatic (AU12) and corrugator (AU4) activity may indicate more positive and more negative subjective intensity, respectively (e.g., [9]). However, later studies found that interactions between multiple AUs better describe valence intensity (e.g., [17]), and in follow-up work, researchers have proposed that the face may represent positive and negative affect simultaneously with independent sets of AUs (e.g., [18]). Of course, the number of AUs that can be simultaneously measured using facial EMG is inherently limited by the number of electrodes that can be used without obstructing the face. Subsequently, facial EMG can only be used to identify a small set of AUs that may be linked to perceived valence intensity. In one of the few studies directly linking AUs to perceived valence intensity, Messinger et al. [19] found that cheek raising (AU6) was common to perceptual judgments of both intense positive and negative affect, which challenges the idea that people may use a single AU to make inference on the entire range of valence intensity. Altogether, current evidence suggests that zygomatic (AU12) and corrugator (AU4) activity indicate perceived positive and negative affect, but the extent to which these and other discrete facial actions map onto the entire range of perceived positive or negative affect intensity is unclear. Note that contemporary theories of emotion propose valence as a core affective state that arises in varying intensity *before* emotional experiences are labelled as happy, sad, etc. [20], suggesting that AUs linked to positive and negative affect are fundamental to the recognition of all other perceived emotions. Therefore, determining the extent to which specific patterns of AUs map to positive and negative affect is important for building on and testing contemporary models of emotion production and recognition.

Comprehensive follow-up investigations have been difficult to pursue, in part, because facial EMG can only detect a very limited number of AUs simultaneously, and manual alternatives are both labor- and time-intensive and require highly skilled annotators. Indeed, FACS training requires an average of 50–100 hours, and minutes of video can take expert coders multiple hours to rate reliably [21]. These characteristics limit sample sizes, reduce feasibility of replication efforts, and discourage researchers from coding facial expressions. Instead, researchers tend to rely on measures of emotional responding that are not observable in social interactions (e.g., heart rate variability). Recently, automated computer-vision and machine learning (CVML) based approaches have emerged that make it possible to scale AU annotation to larger numbers of participants (e.g., [22–24]) thus making follow-up studies more feasible. In fact, inter-disciplinary applications of CVML have allowed researchers to automatically identify pain severity (e.g., [25]), depressive states (e.g., [26]), and discrete emotions from facial expressions (e.g., [27]).

Work using CVML to detect valence intensity from facial expressions is ongoing (see [28]). In fact, there are annual competitions held to develop CVML models that best characterize dimensional features of emotions such as valence and arousal (e.g., [29]). Currently, basic emotions can be coded automatically with accuracy comparable to human coders, but valence intensity models show lower concurrent validity. For example, state-of-the-art CVML models show correlations between human- and computer-coded valence ranging from *r* = .60-.71 [30,31]. While impressive, there are two limitations that have impeded the use of CVML to make inferences on positive and negative affect intensity. Below, we outline each of these limitations and offer our solutions.

First, CVML models are often constructed using difficult to interpret machine learning models that detect valence directly from frame-by-frame video input without intermediately capturing AUs. Therefore, it is both unclear if: (1) successful valence detection depends on prior detection of specific AUs, and (2) machine learning can provide useful insights into how people interpret specific facial actions. In the current study, we show that CVML can be used to both identify well known relationships between AUs and perceived positive and negative affect intensity in addition to revealing novel relationships.

Second, how valence intensity is represented—and therefore measured—varies substantially across studies. For example, some previous CVML models of valence intensity have been developed from relatively small samples or on continuously collected valence ratings (human ratings collected in real-time using dials or joysticks), while others are developed based on static images. It is unclear if such models generalize to other research settings where participants’ emotional expressions to evocative stimuli are coded within discrete, trial-by-trial time intervals (e.g., [32]). Indeed, contemporary work using CVML has shifted from evaluating facial expressions in controlled laboratory settings toward accurately capturing continuous facial expressions of emotion “in the wild”, which is a much more difficult task (e.g., [30,33]). However, given the highly contextual nature of facial expression recognition [20], controlled laboratory settings are ideal for identifying AUs that are specific to perceived core affective processes such as positive and negative affect. Further, most valence-detecting CVML models assume a unidimensional valence continuum as opposed to separable continua for positive and negative affect—to our knowledge, there are few opensource datasets used in CVML research that characterize valence as multi-dimensional (see [34]), and very little work has been done with CVML to separate positive and negative affect (cf. [35]). Notably, positive and negative affect can vary independently and have different predictive values [10,15,36], suggesting that CVML models designed to account for each dimension separately may be most beneficial for behavioral science applications.

Using a well-validated method of emotion induction and both computer-vision measurement of discrete facial actions and continuous measures of positive and negative affect intensity, we (1) identified specific correspondences between perceived emotion intensity and discrete facial AUs, and (2) developed a reliable, valid, and efficient method of automatically measuring the separable dimensions of positive and negative affect intensity. Based on previous work on subjective valence intensity using facial EMG, we hypothesized that CVML would identify AUs 12 and 4 as of the most important AUs for positive and negative affect intensity, respectively. Additionally, we hypothesized that the effects of AUs 12 and 4 on positive and negative affect intensity would depend on the activation of other AUs, and that these interactions could be probed with interpretable machine learning methods. Importantly, data used to train and validate our CVML models were collected from a commonly-used psychological task and contained 4,648 video-recorded, evoked facial expressions from 125 human subjects across multiple task instructions. Our findings shed light on the mechanisms of valence recognition from facial expressions and point the way to novel research applications of large-scale emotional facial expression coding.

## Method

### Participants

Video recordings and human coder data were collected as part of a larger study [32]. The current study included 125 participants (84 females), ages 18–35 years. All participants gave informed consent prior to the study, and the study protocol (#2011B0071) was approved by The Ohio State Behavioral and Social Sciences Institutional Review Board. Self-reported ethnicities of participants were as follows: Caucasian (*n* = 96), East Asian (*n* = 14), African-American (*n* = 5), Latino (*n* = 3), South Asian (*n* = 3), and unspecified (*n* = 4). Note that we tested for racial/ethnic differences in valence coding accuracy, and using Bayesian comparisons we found evidence favoring no differences in accuracy between groups (see Supporting Information).

### Measures

#### Emotion-evoking task

**Fig 1:**
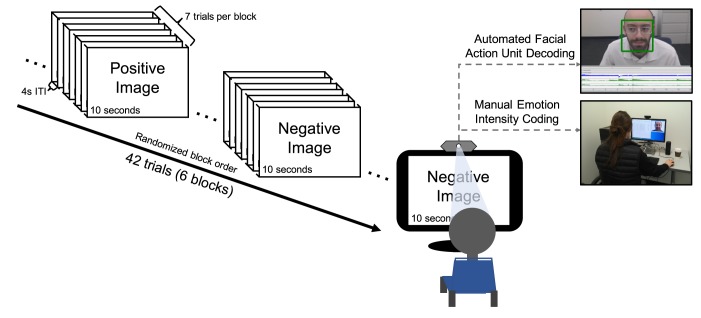
Emotion-evoking task.

We used an emotion-evoking task, depicted in Fig 1, that has been used in several previous studies to elicit facial expressions of emotion across multiple task instructions [32,37]. Participants viewed 42 positive and negative images selected from the International Affective Picture System (IAPS) to balance valence and arousal. Selections were based on previously reported college-student norms [38]. Images were presented in 6 blocks of 7 trials each, whereby each block consisted of all positive or all negative images. For each block, participants were asked to either *enhance*, *react normally*, or *suppress* their naturally evoked emotional expressions to the images. These instructions effectively increased variability in facial expressions within participants. Further, effortful enhancement and suppression of facial expressions is common across many real-world social situations where specific emotional expressions are expected to reach desired outcomes. Given known individual differences in suppression and enhancement of facial expressions [32,37], we expected that these task instructions would allow us to create a more generalizable CVML model than with no instructions at all. Block order was randomized across participants. Instructions were given so that each valence was paired once with each condition. All images were presented for 10 s, with 4 s between each image presentation. Participants’ reactions to each image were video-recorded with a 1080p computer webcam (Logitech HD C270). Due to experimenter error, 1 participant’s videos were not recorded correctly, and 7 participants were shown only 41 recordings, resulting in 6,293 usable recordings. Among these, 3 were corrupted and could not be viewed. Thus, 6,290 10-s recordings were potentially available.

In each of the 3 blocks containing positive and negative image content, participants were asked to either *enhance*, *react normally*, or *suppress* their emotional expressions, so that each valence type (i.e., positive or negative) was paired once with each task instruction (enhance, react normally, suppress). All images were selected from the International Affective Picture System [38]. Participants’ reactions to the images were video recorded and their facial expressions were subsequently rated for positive and negative emotion intensity by a team of trained coders. The same recordings were then analyzed by FACET, a computer vision tool which automatically identifies facial Action Units (AUs). Note that the individual in this figure is of the first author. The individual in this manuscript has given written informed consent (as outlined in PLOS consent form) to publish these case details.

#### Manual coding procedure

A team of three trained human coders, unaware of participants’ task instructions, independently viewed and rated each 10-s recording for both positive and negative emotion intensity. Presentation of recordings was randomized for each coder. Ratings were collected on a 7-point Likert scale ranging from 1 (*no emotion*) to 7 (*extreme emotion*), where positive and negative affect were coded independently following each presentation. Coders completed an initial training phase during which they rated recordings of pre-selected non-study cases and discussed specific facial features that influenced their decisions (see the Supporting Information for the coding guide). The goal of this training was to ensure that all coders could reliably agree on emotion intensity ratings. In addition, coders participated in once-monthly meetings throughout the coding process to ensure reliability and reduce drift. Agreement between coders across all usable recordings (6,290 recordings) was high, with intraclass correlation coefficients (ICCs(3); [39]) of .88 and .94 for positive and negative ratings, respectively. The ICC(3) measure reported above indicates absolute agreement of the average human-coder rating within each condition (*enhance*, *react normally*, *suppress*) for each of the 150 participants in the original study [32]. To prepare data for CVML analysis, we performed an additional quality check to screen out videos in which participants’ faces were off-camera or covered. Any recording in which a participant’s face was covered, obscured, or off-camera for 1 s or more was removed from analysis. If 50% or more of a participant’s recordings were excluded, we excluded all of his/her recordings to ensure that we had enough within-subject data to use for within-subject model performance analyses. This resulted in a total of 4,648 usable recordings across 125 participants. With over 4,000 individually-coded recordings, our sample size is in the typical range for machine learning applications [40].

#### Automated coding procedure

We then analyzed each of the 4,648 recordings with FACET [24]. FACET is a computer-vision tool that automatically detects 20 FACS-based AUs (see S1 Table for descriptions and depictions of FACET-detected AUs). While there are no published validation studies of FACET’s AU detection accuracy to our knowledge, there are many studies validating the Computer Expression Recognition Toolbox (CERT), which is FACET’s opensource predecessor [41]. Validation studies of CERT show that it can discriminate between 18 different AUs with high accuracy rates (e.g., average 2AFC = 80–90%, [41]). Further, FACET has shown better than human accuracy in detecting basic emotions across multiple datasets (e.g., > 95%, [24]), which strongly relies on accurately capturing the AUs that describe each basic emotion category. Note that FACET was recently purchased by Apple Inc. and is no longer available to the public. However, there are other commercial software options available for automated AU detection including Noldus’s FaceReader, Affectiva’s AFFDEX, and the opensource OpenFace package, each of which have been validated in previous studies [22–24]. Importantly, the methodology we use in the current study is not specific to FACET and any of the above software tools could be utilized to replicate our analyses. FACET outputs values for each AU indicating the algorithm’s confidence in the AU being present. Confidence values are output at a rate of 30 Hz, resulting in a time-series of confidence values for each AU being present with each frame of a video-recording. Each point in the time-series is a continuous number ranging from about -16 to 16, whereby more positive and more negative numbers indicate increased and decreased probability of the presence of a given AU, respectively. We refer to this sequence of numbers as an AU evidence time-series.

Each AU evidence time-series was converted to a point estimate by taking the area under the curve (AUC) of the given time-series and dividing the AUC by the total length of time that a face was detected throughout the clip. This creates a normalized measure that does not render biased weights to clips of varying quality (e.g., clips in which participants’ faces are occasionally not detected). Point-estimates computed this way represent the expected probability that a participant expressed a given AU across time. We used the AU evidence time-series point estimates as predictor (independent) variables to train a machine learning model to predict human valence intensity ratings. It took FACET less than 3 days to extract AU evidence time-series data from all recordings (running on a standard 8-core desktop computer). Note that we did not use a baseline correction for each subject, which would require human annotation of a neutral facial expression segment for each participant. Therefore, the models reported here may be applied to novel facial recordings with no human judgment.

In addition to raw AU scores, FACET computes scores for positive and negative affect which reflect the probability that a facial expression is of either positive or negative affect. Although these scores reflect presence of positive or negative affect rather than intensity, we report them alongside our results to emphasize the added predictive validity achieved by our method. We used the same preprocessing steps for FACET’s positive and negative affect scores as for the AUs (i.e. we computed the normalized AUC values for each recording).

### Machine learning procedure

**Fig 2:**
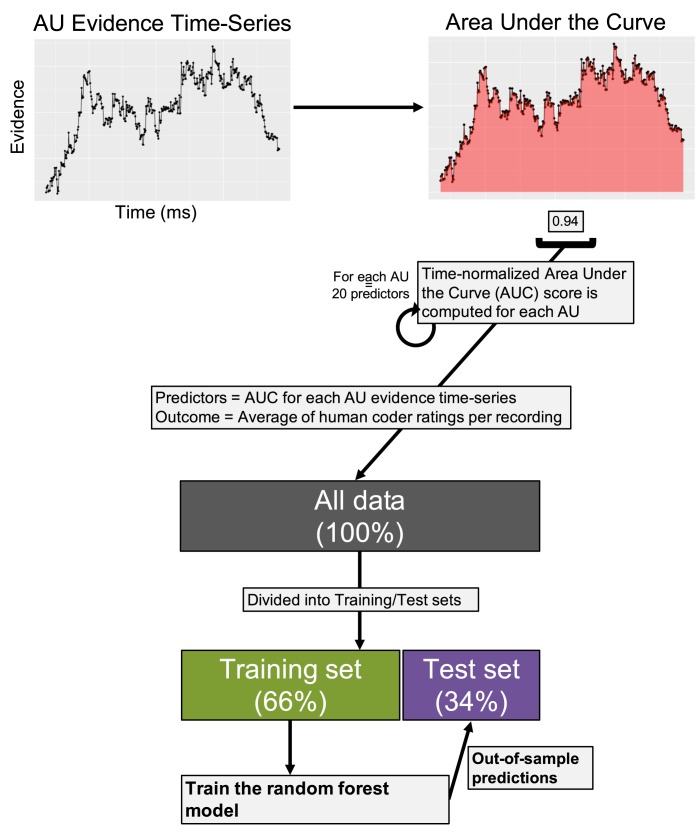
Machine learning procedure. The goal of our first analysis was to determine whether or not CVML could perform similarly to humans in rating facial expressions of emotion. For each AU evidence time-series, we computed the normalized (i.e., divided by the total time that FACET detected a face) Area Under the Curve (AUC), which captures the probability that a given AU is present over time. All AUC values (20 total) were entered as predictors into the random forest (RF) model to predict the average coder rating for each recording. To test how similar the model ratings were to human ratings, we separated the data into training (3,060 recordings) and test (1,588 recordings) sets. We fit the RF to the training set and made predictions on the unseen test set. Model performance was assessed by comparing the Pearson and intraclass correlations between computer- and human-generated ratings in the test sets.

Fig 2 depicts the machine learning procedure. We trained a random forest (RF) model to predict human-coded valence ratings from the AU evidence time-series point estimates described above (see Supporting Information for details on training). RFs are constructed by generating multiple decision trees and averaging predictions of all trees together. We chose the RF model because (1) it can automatically capture interactions between independent variables, and we know that humans use multiple AUs simultaneously when evaluating facial expressions; (2) the importance of each independent variable can be easily extracted from the RF to make inferences regarding which AUs human coders attended to while rating valence intensity (analogous to interpreting *beta* weights from a multiple regression; [40]); and (3) RFs have previously shown robust representations of the mapping from facial features (e.g., AUs) to discrete emotions and valence intensity [42,43]. We additionally tested regularized regression models including the least absolute shrinkage and selection operator (LASSO), ridge regression, and elastic-net, but these linear models did not adequately capture the human ratings. Further, we tested a Deep Neural Network model that performed similarly to the reported RF results (see Supporting Information for model comparison), and due to its ease of use and interpretation we decided to only report the RF model results in the main text .Given high agreement among coders and a large literature showing that aggregating continuous ratings from multiple, independent coders leads to reliable estimates despite item-level noise (i.e., ratings for each recording; see [44]), we used the average of all coders’ ratings for each recording as the outcome (dependent) variable to train the RF.

The RF model contains 2 tuning parameters, namely: (1) *ntrees*–the number of decision trees used in the forest, and (2) *mtry*–the number of predictors to sample from at each decision node (i.e., “split”) in a tree. A grid search over *ntrees* ∈{100, 200, 300,…,1000} showed that out-of-bag prediction accuracy converged by 500 trees for both positive and negative datasets (not reported). A grid search over *mtry* ∈{1, 2, 3,…,20} revealed negligible differences in out-of-bag prediction accuracy for values ranging from 5 to 20. Because RFs do not over-fit the data with an increasing number of trees [40], we set *ntrees* = 500 for models presented in all reported analyses to ensure convergence. Because initial grid searches over *mtry* failed to improve the model, we set *mtry* heuristically [40] as *mtry* = *p*/3, where *p* represents the number of predictors (i.e., 1 for each AU) in an *n* × *p* matrix (*n* = number of cases) used to train the model. We fit the RF model using the *easyml* R package [45], which provides a wrapper function for the *randomForest* R package [46]. All R codes and de-identified data (i.e. FACET output and human coder ratings) used for model fitting along with the trained RF models are available on our lab GitHub, which allow for replication of all analyses and figures (https://github.com/CCS-Lab/Haines_CVML_2018).

#### Correspondence between human coders and model predictions

Model performance refers to how similar the model- and human-generated valence intensity rating are. To assess model performance, we split the 4,648 recordings into training (*n* = 3,060; 65.8%) and test (*n* = 1,588; 34.2%) sets, trained the model on the training set (see the Supporting Information for details), and then made predictions on the unseen test set to assess how well the RF predicted valence intensity ratings on new data. The data were split randomly with respect to participants so that the training and test data contained 66% and 34% of each participant’s recordings, respectively. This separation ensured that training was conducted with all participants, thus creating a more generalizable final model. We fit a separate RF model to positive and negative human ratings. To see if the way we split the training and test data influenced our results, we made 1,000 different training/test-set splits and assessed model performance across all splits [47,48]. We used Pearson correlations and ICC coefficients to check model performance on training- and test-sets. Pearson correlations measure the amount of variance in human ratings captured by the model, whereas ICCs measure absolute agreement between human- and model-predicted ratings at the item level (i.e., per recording). Therefore, high correlations and ICCs indicate the model is capturing a large amount of variance in human coder ratings and generating ratings using a similar scale as human coders, respectively. We used McGraw and Wong’s ICC(1), as opposed to other ICC methods [39], because we were interested in absolute agreement across all clips, regardless of condition/participant. One-way models were used to compute ICCs in all cases. In general, ICCs between .81 and 1.00 are considered “almost perfect” (i.e., excellent) and ICCs between .61 and .80 are considered “substantial” (i.e., good; [49]). We used regression-based approaches and performance measures as opposed to classification-based alternatives (e.g., F1 scores on models trained to classify intensity ratings) because the averaged coder ratings across recordings resembled continuous, real numbers more so than ordinal, categorical intensity scores. Additionally, regression-based models are commonly used in developing models that predict valence and/or arousal intensity. We also checked model performance using a different folding scheme for separating training and test sets which ensured that participants’ recordings were not shared across splits. This analysis revealed negligible differences in prediction accuracy for positive ratings and a decrease in accuracy for negative ratings, which suggests that more training data may be necessary to capture negative as opposed to positive affect intensity (see Supporting Information).

#### Importance of AUs for positive and negative affect

To identify the specific AUs that human coders were influenced most by when making affective ratings, we fit the RF model to the entire dataset (all 4,648 recordings) without splitting into training and test sets. We used this method to identify independent variables that were robust across all samples [47,48]. After fitting the RF models, the importance of each independent variable was estimated using *partial dependence* [50], a measure of the expected standard deviation in the outcome variable (e.g., positive or negative affect intensity) as a function of a given predictor variable (e.g., AU12) averaged across all other predictor variables (e.g., all AUs except AU12). In fact, in special cases, the absolute values of the multiple regression beta weights are equivalent to the corresponding partial dependence metric [50], which makes partial dependence a useful metric for assessing the importance of predictors when using “black-box” methods such as RFs. Crucially, and unlike other methods of measuring variable importance, partial dependence can also be used to probe both directionality and interaction effects when plotted as a function of the model predictors [50].

To determine if CVML could adequately capture the relative importance of AUs for each individual coder, we also fit the RF to each coder’s ratings independently. We used randomization tests to determine the minimum number of ratings necessary to accurately infer which AUs the coders attended to while generating emotion ratings. For each of the 3 coders, we performed the following steps: (1) randomly sample *n* recordings rated by coder *i*, (2) fit the RF model to the subset of *n* recordings/ratings according to the model fitting procedures outlined above, (3) compute the ICC(2) of the extracted RF feature importances (i.e., *partial dependence*) between the subsampled model and the model fit to all recordings/ratings from coder *i*, and (4) iterate steps 1–3 thirty times for each value of *n* (note that different subsets of *n* recordings/ratings were selected for each of these thirty iterations). We varied *n* ∈ {10, 15, 20, 25, 30, 35, 40, 45, 50, 55, 60, 65, 70, 75, 80, 85, 90, 95, 100, 105, 115, 125, 135, 150, 200, 250, 300, 350, 400, 450, 500, 550, 600, 650, 700, 750, 800, 850, 900, 950, 1000, 1200, 1400, 1600, 1800, 2000, 2500, 3000}.

## Results

### Model performance across participants

**Fig 3:**
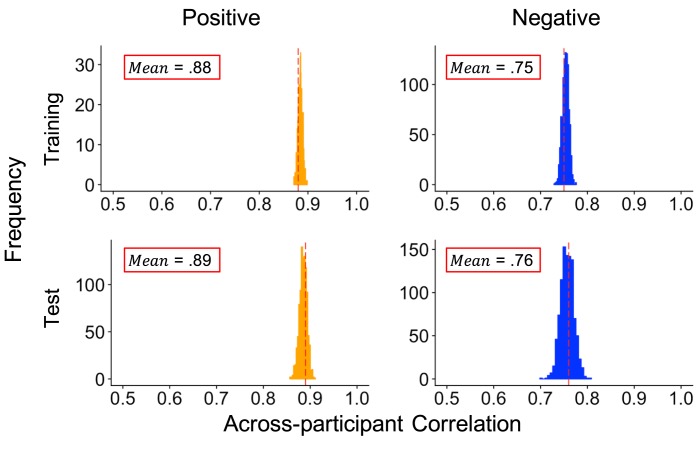
Sensitivity of model performance to different training/test splits. Results of sensitivity analyses across different splits of the training and test sets. We created 1,000 different splits of the training and test sets, fit the RF to each training set, and then made predictions on each respective test set. We stored the Pearson correlations between human- and model-generated ratings for each iteration. Distributions therefore represent uncertainty in prediction accuracy. Means of the distributions (superimposed on respective graphs) are represented by dashed red lines.

Table 1 shows correlations between the model-predicted and the average of the human coders’ ratings per recording across both training and test sets. Overall, the RF showed good to excellent performance across both training and test sets for positive and negative ratings. Notably, these results were supported by both the Pearson correlations and the ICCs, suggesting that the RF produced ratings that not only captured variance in, but also showed high agreement with, human ratings. Sensitivity analyses (see Fig 3) indicated that model performance was robust across different training and test splits of the data. These results suggest that variance in human-coded valence intensity can be captured by the presence of discrete AUs.

**Table 1:** Correlations between human- and computer-generated valence ratings.

Model: Data Set	Correlation [95% CI]			
	*r*		ICC(1)	
	(+)	(–)	(+)	(–)
RF Ratings: Training	.89 [.88, .90]	.77 [.75, .78]	.88 [.87, .89]	.71 [.69, .72]
RF Ratings: Test	.88 [.87, .89]	.74 [.72, .77]	.87 [.86, .88]	.68 [.65, .71]
FACET Ratings: Training + Test	.71 [.70, .73]	.40 [.38, .43]	-.43 [-.46, -.41]	-.22 [-.25, -.20]

*Notes*. (+) = positive valence ratings; (–) = negative valence ratings; *r* = Pearson’s correlation; ICC = Intraclass correlation coefficient. Training and test sets contained 3,060 and 1,588 recordings, respectively. Note that because FACET’s default positive and negative valence scores were not informed by our dataset, we present the correlations of FACET scores across the entire dataset as opposed to separately for training and test sets. ICC(1) scores are not necessarily interpretable for FACET’s positive and negative affect scores because FACET’s scale of measurement is arbitrary (i.e. ranging from about -16 to +16), whereas the human coders made judgements on a meaningful 1–7 scale. Nevertheless, we report them for completeness.

### Model performance within participants

**Fig 4:**
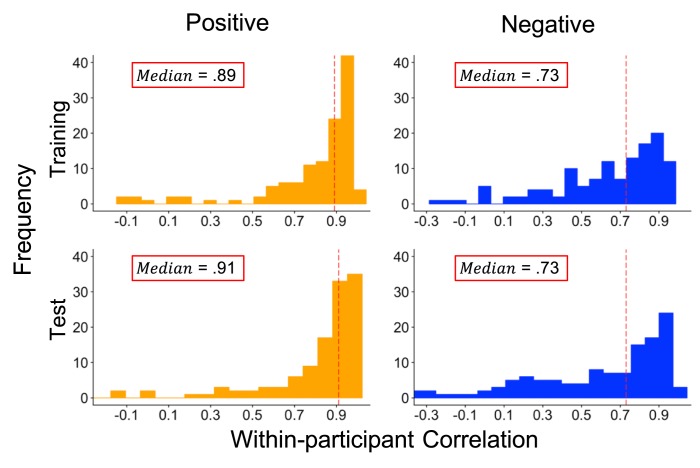
Model performance within participants. Distributions of within-participant Pearson correlations for positive and negative ratings in the training (all 125 participants) and test (122 participants; correlations could not be computed for 3 participants who had 0 variance in human ratings) sets. Red dashed lines represent median within-participant Pearson correlations for each distribution. Intraclass correlations for corresponding figures are reported in text.

We also checked model performance for each of the 125 participants by computing correlations between human- and model-generated ratings for each participant separately (Fig 4). Although the RF model performed well for many participants in the positive (median *r* = .91, ICC(1) = .80) and negative (median *r* = .73, ICC(1) = .51) affect test sets, 5 participants within the positive and 7 participants within the negative affect test-set yielded negative correlations between human- and computer-generated emotion ratings (Fig 4). Further analyses of within-participant model performance revealed significant positive associations between within-subject variance in model-predicted ratings and within-participant prediction accuracy (all *r*s ≥ .54, *p*s < .001; see S2A Fig). We found the same relation between human-assigned ratings and within-participant variance (see S2B Fig). This suggests that the RF model was more accurate in predicting human-rated emotion if participants expressed a wider range of emotional intensity.

### Importance of AUs across task instructions

**Fig 5:**
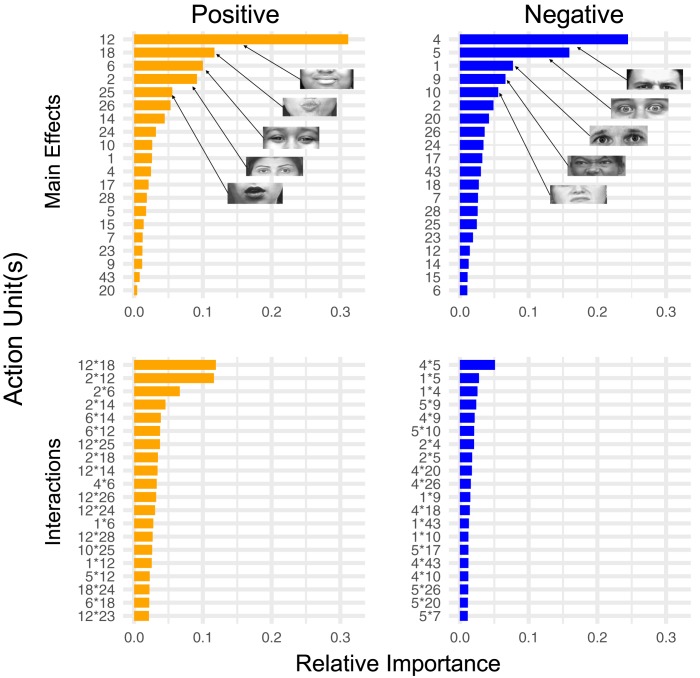
Main and interactive effects among and between AUs for positive and negative ratings. Relative importance of the main effects and interactions among all AUs for positive and negative human-coder ratings. Relative importance (normalized *partial dependence* from the RF model) is a measure the SD in the outcome variable (i.e. positive or negative affect intensity) attributable to each AU while integrating over all other AUs, and it can be interpreted as how important a given AU is with respect to all other AUs. Note that partial dependence is not directional (see Fig 6 for directional effects). Visual depictions of the 5 most important AUs for predicting positive and negative ratings are shown on the graphs. Because there are 190 possible combinations of AUs for displaying interactive effects, we only show the top 20 here for brevity.

To identify which facial expressions human coders may have used to generate positive and negative emotion ratings, we examined the importance of all AUs in predicting human emotion ratings (Fig 5). Note that importance values for the RF do not indicate directional effects, but instead reflect relative importance of a given AU in predicting human-coded positive/negative affect intensity. The RF identified AUs 12 (*lip corner pull*), 6 (*cheek raiser*), and 25 (*lips part*) as three of the five most important AUs for predicting positive emotion. In contrast to positive ratings, relative importance values for AUs of negative ratings were distributed more evenly across AUs, a trend which was also found when the RF was fit individually to each coder (see *Coder-specific AU importance measures* below). Notably, the importance of AUs for positive and negative emotion ratings were largely independent. In fact, when the ICC(3) is computed by treating positive and negative importance weights for each AU as averaged ratings from two “coders”, the ICC(3) is negative and non-significant (ICC(3) = –.48, *p* = .80), which would only be expected if different facial expressions were important for the coders to rate positive versus negative valence. Lastly, the RF identified stronger interactive effects between AUs for positive relative to negative affect intensity (Fig 5). Specifically, interactions between AUs 12*18 and 2*12 together accounted for ~25% of the interactive effects for positive affect, which is exceedingly high given the 190 possible 2-way interactions. Conversely, interactions between AUs for negative affect intensity were more uniformly important, apart from the interaction between AUs 4*5. These differences in interactions between positive and negative affect may be partially attributable to the larger number of possible AU combinations that can indicate negative rather than positive affect.

**Fig 6:**
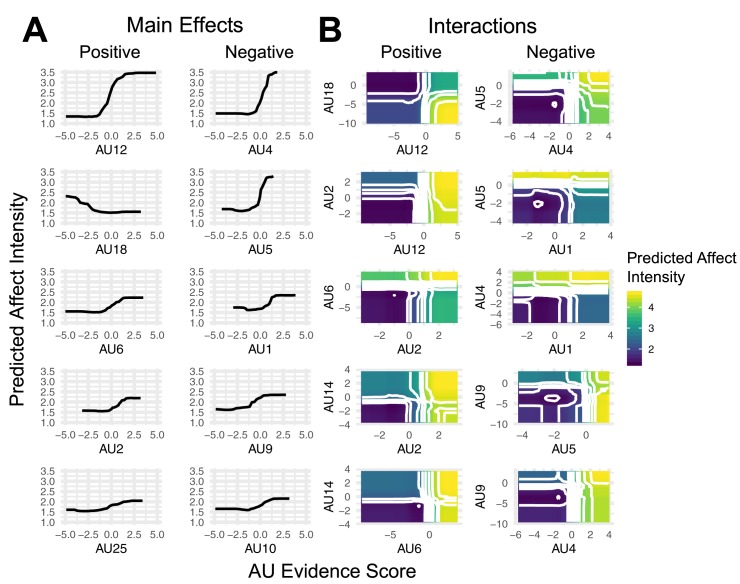
Directionality of main and interactive effects. Partial dependence plots of the 5 most important main and interactive effects for both positive and negative affect intensity ratings. Partial dependence indicates the predicted affect intensity while integrating over all other AUs. Panel (A) shows the directionality of main effects, where increasing (decreasing) values indicate positive (negative) effects as AU presence increases. Panel (B) shows directionality of interactive effects, where warmer (cooler) colors indicate higher (lower) affect intensity ratings given specific combinations of AU presence scores on the x- and y-axes.

The partial dependence analysis measures revealed that the main effects of the 5 most important AUs were in the expected directions for both positive and negative affect intensity ratings (Fig 6). Specifically, AUs 12, 6, and 25 were positively related to increased positive affect intensity, while AUs 4, 5, 9, and 10 were positively related to increased negative affect intensity. Intriguingly, we found that AU18 was negatively related to increased positive affect intensity, which may be attributed to either its masking effects on AU12 or its relation anger. Indeed, the largest interaction for positive affect was between AUs 12 and 18, where high presence scores for AU12 in combination with low presence scores for AU18 predicted high positive affect intensity. For negative affect intensity, we found an interaction between AUs 1 and 5 such that negative affect was most intense when AU5 had high presence scores while AU1 had low presence scores, despite both AUs showing independent, positive relationships with increased negative affect. We found a similar relationship between AUs 5 and 9, which revealed that negative affect was strongest when AUs 5 and 9 had high and low presence scores, respectively. These finding may be attributable to AUs 5 relationship***s*** to fear, surprise, and arousal, of which arousal is often used as an indicator of more intense emotion by human judges (e.g, [51]).

### Sensitivity of AUs to task instructions

To determine if task instructions (*enhance*, *react normally*, *suppress*) affected model performance or our interpretation of which AUs map onto positive and negative affect, we fit the RF model to all recordings from each condition separately and then compared model performance and AU importance scores across conditions. Table 2 shows correlations between human- and computer-generated valence ratings within the different conditions, and summary statistics for AU evidence scores within each condition are provided in S2 Table. For positive ratings, correlations were consistently high (*r*s > .80) across all conditions. In contrast, for negative ratings, correlations were highest in the enhance condition, followed by the react normally and suppress conditions. Of note, all correlations between human- and computer-generated ratings were lower when data were separated by condition compared to when condition was ignored (cf., Table 2 to Table 1). This suggests the lower number of recordings included in the training samples may be partially responsible for lower model performance, but also that CVML performs best when trained on a wider range of emotional intensity. Indeed, our supplementary analyses showed that when participants had lower variance in affect intensity (determined by either human or model ratings), the correspondence between human and model ratings tended to be lower as well (see S2 Fig). This finding suggests that lower model performance in the Suppression condition may be due to limited variation in human ratings for the model to predict.

**Table 2:** Correlations between human- and computer-generated ratings within conditions.

Condition	Correlation [95% CI]				Number of recordings	
	*r*		ICC(1)		Training	Test
	(+)	(–)	(+)	(–)		
Enhance	.81 [.78, .84]	.64 [.59, .68]	.79 [.76, .82]	.61 [.55, .66]	1,047	569
Normal	.81 [.78, .84]	.55 [.49, .61]	.79 [.76, .82]	.49 [.42, .55]	880	516
Suppress	.85 [.83, .87]	.44 [.38, .51]	.83 [.80, .85]	.35 [.28, .42]	1,040	596

*Notes*. (+) = positive valence ratings; (–) = negative valence ratings; *r* = Pearson’s correlation; ICC = Intraclass correlation coefficient. All results reported are on test sets.

**Fig 7:**
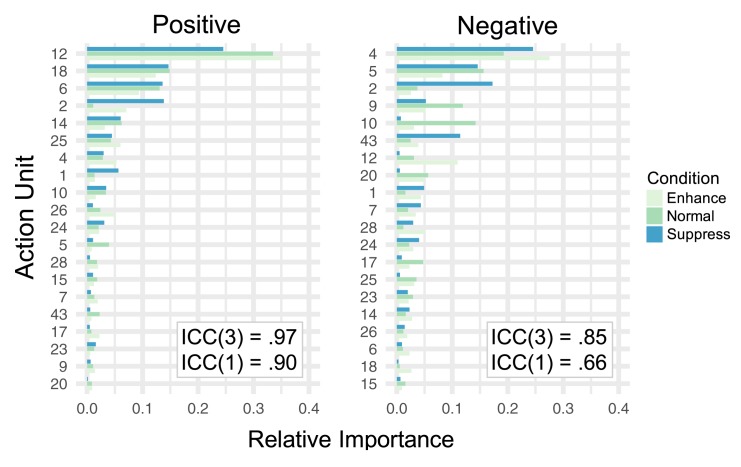
AU relative importance values across task instructions. Relative importance of each AU for positive valence and negative valence human-coder ratings within each of the three task instructions (*enhance*, *react normally*, *suppress*). Intraclass correlation coefficients–both treating importance values as average [ICC(3)] and single [ICC(1)] units–are superimposed. We show ICC(3) here because the AU importance scores could be interpreted as “averages” across all recordings.

Despite only moderate correlations for negative ratings in these conditions, relative importance values for AUs across conditions showed minimal differences (Fig 7). In fact, ICCs between AU importance values across conditions were excellent for both positive and negative ratings (Fig 7). Taken with our supplementary analysis of variation in human ratings and model performance, these results suggest that the task instructions did not strongly influence the interpretation of important AUs for detecting positive and negative affect intensity across coders.

### Coder-specific AU importance measures

All three coders showed similarly-ordered importance profiles, indicating that they attended to similar AUs while generating emotion ratings (S3 Fig). Agreement between all three individual coders’ importance profiles supported this claim—non-normalized ICC(3)s were high for both positive (ICC(3) = 0.93) and negative (ICC(3) = 0.90) importance profiles. The randomization test revealed how many recordings were necessary to adequately estimate the relative importance of AUs for each individual coder. For positive ratings, ICC(2)s for all 3 coders reached 0.75 (regarded as “excellent” agreement; see 39) after approximately 60 recordings/ratings. For negative ratings, ICC(2)s for all 3 coders reached 0.75 after approximately 150 recordings/ratings (see S4 Fig). Because the recordings in our task were 10 s long and coders rated positive/negative emotion intensity after each recording, the task used in the current study could be condensed to about 150 recordings (<30 minutes) and still reveal coder-specific AU importance measures with good accuracy. Future studies may be able to shorten the task even further by testing shorter video recordings (i.e., less than 10 s per recording).

## Discussion

Our study offers strong evidence that people use discrete AUs to make wholistic judgments regarding positive and negative affect intensity from facial expressions, indicating that patterns of discrete AUs reliably represent dimensions of facial expressions of emotion (analogous to how specific patterns of AUs map to the basic emotions). Our CVML analysis identified AU12, AU6, and AU25 as especially important features for positive affect intensity ratings. Together, these AUs represent the core components of a genuine smile [52]. Note that AU12 and AU6 interact to signify a *Duchenne smile*, which can indicate genuine happiness [8], and previous research demonstrates that accurate observer-coded enjoyment ratings rely on AU6 [53]. Additionally, the five most important AUs we identified for negative affect intensity map on to those found in negative, discrete emotions such as fear and anger (AUs 4 and 5), disgust (AU9), and sadness (AU4). While AU12 and AU4 have been implicated in positive and negative affect for some time (e.g., [9]), this is the first study of its kind to determine the relative importance of these and other AUs in determining positive and negative affect intensity. Importantly, the strong correspondence that we found between specific sets of AUs and positive and negative valence intensity suggests that contemporary models of constructed emotion may be further tested against basic emotion theories in experimental settings. For example, future studies may investigate the time course of facial expression detection, where basic versus constructed emotion theories make differential predictions on whether basic emotional categories versus emotional dimensions are recognized more accurately and/or rapidly.

Together, the AUs that we identified for positive and negative affect are consistent with prior studies suggesting that positive and negative facial expressions occupy separate dimensions [15,54]. Notably, the AUs accounting for the majority of the variance in positive affect had no overlap with those for negative affect, evidenced by near-zero ICCs, indicating that our human coders used distinct patterns of facial expressions to evaluate positive versus negative intensity ratings. The existence of distinct patterns of AUs which represent positive and negative affect intensity explains paradoxical findings that facial expressions can be simultaneously evaluated as both positive and negative (e.g., happily-disgusted; [10]). Importantly, prior studies have shown that automated facial expression recognition tools such as FACET sometimes fail to recognize blended expressions as accurately as human observers do, which is in part human observers rely strongly on affective valence whereas tools such as FACET rely on morphological features when making classifying expressions (e.g., AUs; [55]). Our results suggest that this inherent limitation of automated tools can potentially be overcome if morphological features are used to train models to predict valence intensity, which may then allow CVML to make better distinctions between prototypical and blended facial expressions. Further, our supplementary results suggest that the use of CVML to determine the relative importance of AUs for positive and negative affect recognition within individual coders is a potentially important avenue for future research. While the current study only determined relative AU importance for three trained coders (see S3 and S4 Figs), future studies may collect emotion ratings from larger, naïve groups of participants and perform similar analyses to assess for potential individual differences.

Our results also provide support for the use of CVML as a valid, efficient alternative to human coders, and with further validation we expect CVML to expand the possibilities of future facial expression research in the social and behavioral sciences. For example, adoption of automatic facial coding tools will allow researchers to more easily incorporate facial expressions into models of human decision making. Decades of research show clear links between facial expressions of emotion and cognitive processes in aggregate (see [56,57]), yet the dynamics between cognitive mechanisms and facial expressions are poorly understood in part due to difficulties accompanying manual coding. In fact, we are currently using computational modeling to explore cognition-expression relationships with the aid of CVML [58], which would be infeasible with manual coding of facial expressions. For example, in the current study it took less than three days to automatically extract AUs from 4,648 video recordings and train ML models to generate valence intensity ratings (using a standard desktop computer). In stark contrast, it took six months for three undergraduate human coders to be recruited, trained, and then code *affect intensity* across our 125 subjects—FACS coding would have taken much longer, rendering the scale of this project infeasible.

Models used in this study predicted positive emotion intensity with greater accuracy than negative emotion intensity, which may be due to the number of discrete facial actions associated with negative compared to positive emotional expressions. To support this claim, we found that importance scores for negative, but not positive, emotion ratings were spread across many different AUs and showed more variation across task instructions (Figs 5 and 7). This suggests that a wider range of facial expressions were used by coders when generating negative rather than positive emotion ratings. Future studies might address this with CVML models that can detect more than the 20 AUs used here. Additionally, our results suggest that negative affect intensity requires more training data for CVML than positive affect, as evidenced by large discrepancies in model performance between our CVML model that ignored the task instructions compared to those that we fit to data from each task instruction separately. Future studies might address this by devoting more time to collecting and coding negative, rather than positive, affective facial expressions.

Our interpretation of the computer-vision coded AUs in this study is potentially limited because we did not compare reliability of AU detection between FACET and human FACS experts. Additionally, FACET only detects 20 of the approximately 33 AUs described by FACS, so it is possible that there were other important AUs to which the human coders attended when generating valence ratings that we were unable to capture. However, our models showed excellent prediction accuracy on new data (i.e., capturing ~80% of the variance in human ratings of positive affect intensity), and we identified theoretically meaningful patterns of AUs for positive and negative emotion intensity that are consistent with prior studies (e.g., components of the *Duchenne smile*). Crucially, of the AUs that were identified as important for positive and negative affect intensity, our interpretable machine learning analyses revealed that each AU had main and interactive effects that were in the theoretically predicted directions (e.g., AU12 and AU4 predicting increased positive and negative affect intensity, respectively). It is unlikely that we would achieve these results if FACET did not reliably detect similar, important AUs which represented the intensity of positive and negative facial expressions produced by our 125 participants. Further, because FACET is intended for commercial use, it has been trained on a large number of participants across a variety of different genders, ages, and ethnicities, which is likely why our model generalized well across ethnicities despite our predominantly Caucasian sample (see Supporting Information). Finally, as computer vision advances, we expect that more AUs will be easier to detect. CVML provides a scalable method that can be re-applied to previously collected facial expression recordings as technology progresses. Our interpretation of the relative importance of AUs for perceptual ratings of positive and negative affect intensity is clearly limited by our relatively low number of coders. However, the strong correspondence we found between human- and model-predicted affect intensity is made stronger by the number of subjects and recordings per subject used to train our models, and our supplementary analyses showed that our design may be expanded to larger numbers of “coders” (i.e. participants) with a substantially reduced number of recordings to empirically probe coder-specific AU importance measures for positive and negative affect intensity recognition (see S4 Fig).

Although this study investigated positive and negative affect, our method could easily be extended to identify facial actions that are associated with other emotional constructs (e.g., arousal). The ability to identify specific AUs responsible for facial expression recognition has implications for various areas within the social and behavioral sciences. Opportunities may be particularly pronounced for psychopathology research, where deficits and/or biases in recognizing facial expressions of emotion are associated with a number of psychiatric disorders, including autism, alcoholism, and depression [59–61]. CVML provides a framework through which both normal and abnormal emotion recognition can be studied efficiently and mechanistically, which could lead to rapid and cost-efficient markers of emotion recognition in psychopathology [62].

## Supporting information

S1 Fig Sensitivity of model performance to different training scheme. Test set performance for the RF model fit using 1,000 training/test splits where separate participants were used to train and test the model. Note that performance for positive affect intensity—but not negative affect intensity—is indistinguishable from results reported in the main text (c.f. Fig 3), suggesting that models of negative affect intensity may require a more diverse set of training data (i.e. more participants) compared to positive affect intensity.
(EPS)

S2 Fig Probing within-participant model performance. (A) Pearson’s correlations between within-participant model performance (Pearson’s *r*; see Fig 4) and the logarithm of within-participant human rating standard deviation (*SD*). Human-rated *SD*s were computed as the logarithm of the *SD* of human coders’ ratings across a given participants’ recordings. Cases with zero variance in human ratings (i.e., all ratings were “1”) are excluded from this analysis. Correlations and the number of participants included in each comparison are superimposed on their respective graphs. All correlations are significant (*p*s < 0.001). (B) Pearson’s Correlations between within-participant model performance (see Fig 4) and the logarithm of within-participant computer rating standard deviation. Computer-rated *SD*s were computed in the same way as human-rated *SD*s, but the model estimates were used in place of the true human ratings. All correlations are significant (*p*s < 0.001).
(EPS)

S3 Fig Coder-specific AU importance measures. Partial dependence scores (not normalized to show relative differences) extracted from the RF model fit separately to each coder. Coders all show similarly ordered importance profiles, suggesting that they attended to similar facial expressions while generating emotion ratings. Note that positive importance estimates are distributed across fewer predictors (i.e., AUs 6, 12, and 18), whereas negative importance estimates are more spread out throughout all predictors. Agreement between all three individual coders’ importance profiles was high, with ICC(3)s of .93 and .90 for positive and negative ratings, respectively.
(EPS)

S4 Fig Number of recordings necessary to accurately estimate AU importance. Grid searches over the number of recordings/ratings necessary to achieve reliable estimates of AU importances for each valence-coder pair (coders appear in the same order as in S3 Fig). Reliability is indexed by the ICC(2) between AU importance profiles (i.e. *partial dependence*) extracted from the model fit to all the recordings that coders rated versus the model fit to subsets of recordings that they rated. Note that the ICC(2) assumes that importance estimates are “average” units (similar to ICC(3)s in Fig 6). The RF model was fit to each sample of size *n* along the *x*-axis, AU importance profiles were extracted from the model, and ICC(2)s were then calculated between the given sample and full-data AU importance profile scores. We iterated this procedure 20 times within each different sample size to estimate the variation in estimates across recordings. Shading reflects the 2 standard errors from the mean ICC within each sample across all 30 iterations. The red-dashed line indicates an ICC(2) of .75, which is considered “excellent”. For positive ratings, the ICC(2) reached .75 after ~60 recordings/ratings for each coder. For negative ratings, all coders reached an ICC(2) of .75 by ~150 recordings/ratings.
(EPS)

S5 Fig Regularized regression model performance. Results of the Elastic Net with various settings for α (including the LASSO at α = 1 and Ridge Regression at α = 0). Distributions shown are generated in the same way as those in Fig 3. Model performance was not affected by changes in α, thus, the LASSO model was selected and compared against the RF model.
(EPS)

S6 Fig Deep neural network model performance. Performance of the DNN in both training and test sets across a grid of different numbers of hidden layers and nodes per hidden layer. Note that the RF model performed similarly to the DNN across all the values within the grid.
(EPS)

S1 Table Facial action units detected by FACET. *Note*. Pictures and descriptions of all Action Units used in the current study. Images were adapted from https://www.cs.cmu.edu/~face/facs.htm.
(PDF)

S2 Table Average evidence scores for action units within conditions. (PDF)

S1 Supporting Information (DOCX)
