# Correction: Using computer-vision and machine learning to automate facial coding of positive and negative affect intensity

**DOI:** 10.1371/journal.pone.0213756

**Published:** 2019-03-07

**Authors:** The PLOS ONE Staff

There are errors in the Funding statement. The publisher apologizes for the errors. The correct Funding statement is as follows: This research was supported by the Basic Science Research Program through the National Research Foundation of Korea (NRF) funded by the Ministry of Science, ICT, & Future Planning (2018R1C1B3007313).
