# Supplementary material for: Using computer-vision and machine learning to automate facial coding of positive and negative affect intensity
Source: PLoS One. 2019 Feb 5;14(2):e0211735. doi: 10.1371/journal.pone.0211735 (PMC6363175; doi:10.1371/journal.pone.0211735)
Supplement: S1 Table — Note. Pictures and descriptions of all Action Units used in the current study. Images were adapted from https://www.cs.cmu.edu/~face/facs.htm. (PDF) [file pone.0211735.s007.pdf]

S1 Table. Facial Action Units Detected by FACET

Action Unit	Explanation	Example
**1**	Inner Brow Raiser	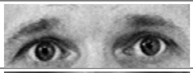
**2**	Outer Brow Raiser	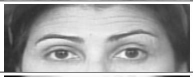
**4**	Brow Lowerer	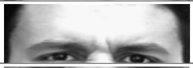
**5**	Upper Lid Raiser	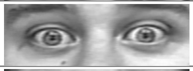
**6**	Cheek Raiser	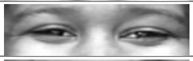
**7**	Lid Tightener	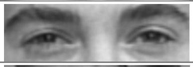
**9**	Nose Wrinkler	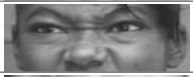
**10**	Upper Lip Raiser	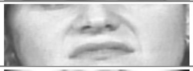
**12**	Lip Corner Puller	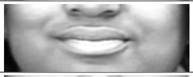
**14**	Dimpler	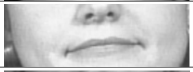
**15**	Lip Corner Depressor	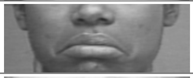
**17**	Chin Raiser	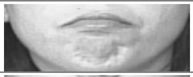
**18**	Lip Puckerer	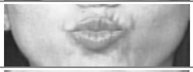
**20**	Lip stretcher	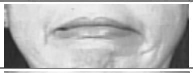
**23**	Lip Tightener	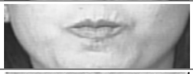
**24**	Lip Pressor	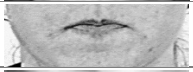
**25**	Lips part	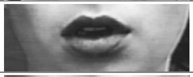
**26**	Jaw Drop	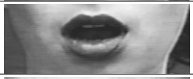
**28**	Lip Suck	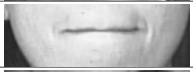
**43**	Eyes Closed	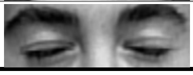

Note. Pictures and descriptions of all Action Units used in the current study. Images were adapted from https://www.cs.cmu.edu/~face/facs.htm.
