# Supplementary material for: Using computer-vision and machine learning to automate facial coding of positive and negative affect intensity
Source: PLoS One. 2019 Feb 5;14(2):e0211735. doi: 10.1371/journal.pone.0211735 (PMC6363175; doi:10.1371/journal.pone.0211735)
Supplement: S2 Table — (PDF) [file pone.0211735.s008.pdf]

S2 Table. Average Evidence Scores for Action Units Within Conditions

Action Unit	Condition (Mean [SD])		
	Enhance	Neutral	Suppress
1	-0.40 (0.86)	-0.65 (0.65)	-0.68 (0.61)
2	-0.59 (0.81)	-0.74 (0.62)	-0.70 (0.60)
4	-0.56 (0.96)	-0.75 (0.75)	-0.83 (0.66)
5	-1.51 (0.72)	-1.70 (0.61)	-1.65 (0.65)
6	-0.93 (1.01)	-1.24 (0.86)	-1.63 (0.74)
7	-0.62 (0.72)	-0.76 (0.63)	-0.86 (0.61)
9	-2.42 (1.29)	-2.54 (1.20)	-2.83 (1.11)
10	-0.70 (0.83)	-0.92 (0.81)	-1.23 (0.70)
12	-1.00 (1.34)	-1.39 (1.06)	-2.06 (0.79)
14	-0.74 (0.77)	-0.84 (0.72)	-1.27 (0.60)
15	-1.19 (0.73)	-1.22 (0.68)	-1.27 (0.70)
17	-0.50 (0.69)	-0.53 (0.59)	-0.65 (0.63)
18	-1.05 (1.40)	-0.65 (0.85)	-0.46 (0.81)
20	-1.28 (0.69)	-1.40 (0.62)	-1.61 (0.59)
23	-0.63 (0.58)	-0.65 (0.54)	-0.80 (0.54)
24	-0.57 (0.77)	-0.56 (0.63)	-0.84 (0.63)
25	-1.66 (1.18)	-1.94 (0.85)	-1.98 (0.80)
26	-1.62 (0.88)	-1.76 (0.76)	-1.79 (0.75)
28	-2.37 (1.16)	-2.50 (1.02)	-3.08 (0.95)
43	-0.75 (1.19)	-0.85 (1.17)	-1.05 (1.24)
