# Supplementary material for: Using computer-vision and machine learning to automate facial coding of positive and negative affect intensity
Source: PLoS One. 2019 Feb 5;14(2):e0211735. doi: 10.1371/journal.pone.0211735 (PMC6363175; doi:10.1371/journal.pone.0211735)
Supplement: S1 Supporting Information — (DOCX) [file pone.0211735.s009.docx]

**Supporting Information**

**Sensitivity of Model Performance to Train/Test Splitting Scheme**

To evaluate model performance when participants’ recordings were not shared across training and test sets, we repeated the model fitting procedure for both positive and negative affect intensity models while splitting the training and test such that 66% and 34% of the participants were used to train and test the model, respectively. Because this method does not train the model to 100% of participants, we expected that model performance would be lower as compared to the results reported in the main text. Model performance for both positive and negative affect intensity was lower using this folding scheme, although the performance loss for positive affect intensity was negligible (S1 Fig).

**S1 Fig. Sensitivity of model performance to different training scheme**

Test set performance for the RF model fit using 1,000 training/test splits where separate participants were used to train and test the model. Note that performance for positive affect intensity—but not negative affect intensity—is indistinguishable from results reported in the main text (c.f. Fig 3), suggesting that models of negative affect intensity may require a more diverse set of training data (i.e. more participants) compared to positive affect intensity.

**Human Coder Guide**

The following guide was developed during the initial training phase (i.e., when coders were rating non-study cases) to aid coders in maintaining high agreement. Note that the coders played a primary role in developing the guide.

1. Flat mouth, bored eyes throughout most of the clip
2. Slight smirk/frown + no eye change held for half clip, or

eyes widening/eyebrows change + no mouth change held for half clip

1. Slight smirk/frown + no eye change held for whole clip, or

eyes widening/eyebrows change + no mouth change held for whole clip

(a.k.a. *Mona Lisa* smile)

1. Smirk/frown with some eye change; may be ambiguous but involves whole face; held for half clip
2. Clear smile/frown with some eye change; mouth may be open for positive emotion
3. Clear smile/frown with eye change; mouth may be open for negative emotion
4. Obvious and definite emotion; held for most of the clip. If you would call them bubbly, excited, forlorn, or terrified

**Analysis of Within-participant Model Performance**

We found a significant positive association between within-participant (log) standard deviation (*SD*) in human- and computer-generated ratings and within-participant model performance (i.e., predicted versus actual rating correlations; S2 Fig). This relation was found in positive and negative training and test sets, suggesting that the RF model performs better as subjects express a wider range of emotional intensity. These results suggest that within-participant *SD* in computer-generated ratings may serve as a marker for identifying participants whose facial expressions are not well identified by ML models. In future applied studies using automated rating tools, within-participant *SD* may be useful in screening for participant whose facial expressions need to be scored/verified by human coders.

**S2 Fig. Probing within-participant model performance**

(A) Pearson’s correlations between within-participant model performance (Pearson’s *r*; see Fig. 4) and the logarithm of within-participant human rating standard deviation (*SD*). Human-rated *SD*s were computed as the logarithm of the *SD* of human coders’ ratings across a given participants’ recordings. Cases with zero variance in human ratings (i.e., all ratings were “1”) are excluded from this analysis. Correlations and the number of participants included in each comparison are superimposed on their respective graphs. All correlations are significant (*p*s < 0.001). (B) Pearson’s Correlations between within-participant model performance (see Fig. 4) and the logarithm of within-participant computer rating standard deviation. Computer-rated *SD*s were computed in the same way as human-rated *SD*s, but the model estimates were used in place of the true human ratings. All correlations are significant (*p*s < 0.001).

**Alternative Machine Learning Models**

**Least Absolute Shrinkage and Selection Operator (LASSO)**

LASSO is a penalized regression model with automated variable selection [1]. The LASSO model imposes an *L1* penalty term on the model, which penalizes the sum of the absolute values for *beta* weights of all predictors; this constraint effectively shrinks all *beta* weights by a constant amount. Unimportant coefficients (i.e., predictors that do not account for much variance in the dependent variable) are shrunk to zero, and surviving coefficients are used for inference. This procedure reduces the chance of overfitting and simplifies interpretation, as fewer parameters are used in the final model. The LASSO model is a special case of the Elastic Net [2], which imposes both *L1* and *L2* penalty terms on the model. We fit the LASSO model using the *easyml* R package [3].

**Deep Neural Network (DNN)**

The (feedforward) DNN model is a graphical model composed of multiple layers of regression models [4] with the goal of learning the parameters *θ* that result in the best approximation of function $f$^*^ for some mapping $y=f\left( x \right| \theta)$. Each hidden layer, as well as the final layer, in the DNN passes through a nonlinear activation layer or transfer function. In the current study, we chose a Rectified Linear Unit (ReLU) function as the activation layer. The DNN model was fit using the *keras* Python package [5], which provides wrapper functions for the *Tensorflow* Python package [6].

**Parameter Tuning**

The LASSO model contains a single tuning parameter$\lambda(\lambda\geq0)$, which is the effective degrees of freedom for the model–as $\lambda$ approaches zero, the model approaches a non-penalized multiple regression model. We tuned $\lambda$ using 10-fold cross-validation. General *k*-fold cross-validation follows these steps: (1) the training data is split into *k* different folds, (2) *k*–1 folds are used to fit the model, (3) predictions are made on the left-out fold, (4) an out-of-sample prediction error is calculated on the left-out fold, and (5) steps 2 through 4 are repeated until each of *k* folds has been left out. The mean squared error over all *k* folds is minimized by a grid search over various values for $\lambda(\lambda\geq0)$. To estimate *beta* weights, the above cross-validation steps were iterated 1,000 times. Values of survived (i.e., not shrunk to zero) *beta* weights were recorded for each iteration. Confidence intervals for each beta weight were calculated based on the variation of the *beta* weight estimate across cross-validation iterations. The above steps are described in extensive detail in a previous study [7]. Because the LASSO is a special case of the Elastic Net where the mixing parameter is set to 1 ($\alpha=1$), we tested the Elastic Net (S5 Fig) with varying values for $\alpha\in\{0.0, 0.2, 0.4, 0.6, 0.8, 1.0\}$. Results showed that the LASSO penalty ($\alpha=1$) was sufficient.

The DNN model used in the current study contains 2 tuning parameters, namely: (1) *n_hidden_layers* – the number of hidden layers used in the network, and (2) *n_hidden_nodes* – the number of nodes in each hidden layer. A grid search (S6 Fig) over *n_hidden_layers* $\in\left\{ 2, 3, 4, 5, 6, 7, 8 \right\}$ showed that out-of-sample prediction accuracy converged by 4 hidden layers for the positive dataset and 7 hidden layers for the negative dataset. A grid search (S6 Fig) over *n_hidden_nodes* $\in\left\{ 256, 512, 1024 \right\}$ revealed negligible differences in out-of-sample prediction accuracy for values ranging from 512 to 1,024, so we set *n_hidden_nodes* = 512 for the final DNN model which was compared to competing models below.

**Across- and Within-subject Test Set Model Performance**

Across subjects, the DNN showed high correlations for positive (*r* = 0.87; ICC(1) = 0.87) and negative (*r* = 0.76; ICC(1) = 0.68) affect intensity, and the LASSO model showed the lowest–although still high–correlations for positive (*r* = 0.84; ICC(1) = 0.83) and negative (*r* = 0.65; ICC(1) = 0.59) ratings.

To compare models, we conducted paired *t*-tests on within-subject (Pearson’s) correlations between human- and model-predicted positive and negative affect intensity ratings in the test sets. Comparing models in this fashion allowed us to determine which model accounted for the most within-subject human-rating variance. The RF outperformed the LASSO for the negative (RF–LASSO: *t*(118) = 2.69, 95% CI = [0.02, 0.14], *p* = 0.008), but not positive (RF–LASSO: *t*(116) = 1.67, 95% CI = [-0.008, 0.10], *p* = 0.10) emotion ratings. Unlike the RF, the DNN failed to outperform the LASSO model for both positive (DNN–LASSO: *t*(115) = 1.24 95% CI = [-0.03, 0.13], *p* = 0.22) and negative (DNN–LASSO: *t*(117) = 1.08, 95% CI = [-0.04, 0.13], *p* = 0.28) emotion ratings. The RF and DNN failed to show significant differences for both positive (RF–DNN: *t*(117) = -0.46, 95% CI = [-0.10, 0.06], *p* = 0.64) and negative (RF–DNN: *t*(118) = 0.83, 95% CI = [-0.05, 0.12], *p* = 0.41) emotion ratings. Together, the within-subject model performance comparisons suggest that the RF model can predict human emotion ratings with equivalent or greater accuracy than the LASSO or DNN. We decided to report only the RF results in the main text for brevity.

**Comparison of RF Model Performance Across Ethnicities**

To test if our largely Caucasian (*n* = 93) sample biased predictions made within other ethnic groups (*n* = 27), we conducted Bayesian independent samples *t*-tests on within-subject model performance (i.e. correlation between human-rated and computer-predicted emotion intensity ratings) between Caucasians and other ethnicities. Bayesian analyses were carried out using JASP [8,9], an open-source toolkit for applying Bayesian statistics. We used Bayesian methods because they allowed us to interpret evidence in favor of a null effect (i.e., no group differences), which cannot be achieved using traditional frequentist methods [10]. We used Bayes Factors to interpret model evidence. Because Bayes Factors are computed using differences between prior and posterior distributions, the choice of prior is important to interpret the end result. We assumed that the differences between groups were distributed along a Cauchy distribution centered at zero with a width of 0.707; the width represents the interquartile range, so 0.707 translates to a 50% confidence that the true effect size (i.e. the true difference between Caucasians and other ethnicities) lies between -0.707 and 0.707. This particular parameterization of the Cauchy width assigns more prior probability to a zero-effect than a uniform distribution does, thus requiring more posterior evidence in favor of the null hypothesis to generate large Bayes Factors [11]. The analysis revealed moderate evidence in favor of the null hypothesis in both training (Bayes Factors of 4.3 and 4.4 for positive and negative ratings, respectively) and test sets (Bayes Factors of 3.5 and 4.3 for positive and negative ratings, respectively). The Bayes Factors show that the differences in model performance we found between Caucasians and other ethnicities are about 3.5-4.4 times more likely under a model assuming no group differences than one assuming differences. Taken together, these results suggest that our largely Caucasian sample did not substantially bias computer-rated emotion intensities made within different ethnic groups, which may be attributable to the large and diverse set of training data leveraged by FACET to generate AU presence ratings.

**S3 Fig. Coder-specific AU importance measures**

Partial dependence scores (not normalized to show relative differences) extracted from the RF model fit separately to each coder. Coders all show similarly ordered importance profiles, suggesting that they attended to similar facial expressions while generating emotion ratings. Note that positive importance estimates are distributed across fewer predictors (i.e., AUs 6, 12, and 18), whereas negative importance estimates are more spread out throughout all predictors. Agreement between all three individual coders’ importance profiles was high, with ICC(3)s of .93 and .90 for positive and negative ratings, respectively.

**S4 Fig. Number of recordings necessary to accurately estimate AU importance**

Grid searches over the number of recordings/ratings necessary to achieve reliable estimates of AU importances for each valence-coder pair (coders appear in the same order as in S3 Fig). Reliability is indexed by the ICC(2) between AU importance profiles (i.e. *partial dependence*) extracted from the model fit to all the recordings that coders rated versus the model fit to subsets of recordings that they rated. Note that the ICC(2) assumes that importance estimates are “average” units (similar to ICC(3)s in Fig 6). The RF model was fit to each sample of size *n* along the *x*-axis, AU importance profiles were extracted from the model, and ICC(2)s were then calculated between the given sample and full-data AU importance profile scores. We iterated this procedure 20 times within each different sample size to estimate the variation in estimates across recordings. Shading reflects the 2 standard errors from the mean ICC within each sample across all 30 iterations. The red-dashed line indicates an ICC(2) of .75, which is considered “excellent”. For positive ratings, the ICC(2) reached .75 after ~60 recordings/ratings for each coder. For negative ratings, all coders reached an ICC(2) of .75 by ~150 recordings/ratings.

**S5 Fig. Regularized regression model performance**

Results of the Elastic Net with various settings for 𝛼 (including the LASSO at 𝛼 = 1 and Ridge Regression at 𝛼 = 0). Distributions shown are generated in the same way as those in Fig 3. Model performance was not affected by changes in 𝛼, thus, the LASSO model was selected and compared against the RF model.

**S6 Fig. Deep Neural Network model performance**

Performance of the DNN in both training and test sets across a grid of different numbers of hidden layers and nodes per hidden layer. Note that the RF model performed similarly to the DNN across all the values within the grid.

**S1 Table. Facial Action Units Detected by FACET**

*Note*. Pictures and descriptions of all Action Units used in the current study. Images were adapted from <https://www.cs.cmu.edu/~face/facs.htm>.

**S2 Table. Average Evidence Scores for Action Units Within Conditions**

**Supporting Information References**

1. Tibshirani R. Regression shrinkage and selection via the lasso. Journal of the Royal Statistical Society. Series B (Methodological). 1996 Jan 1:267-88.
2. Zou H, Hastie T. Regularization and variable selection via the elastic net. Journal of the Royal Statistical Society: Series B (Statistical Methodology). 2005 Apr 1;67(2):301-20.
3. Ahn WY, Hendricks P, Haines N. Easyml: Easily Build and Evaluate Machine Learning Models. bioRxiv. 2017.
4. Goodfellow I, Bengio Y, Courville A, Bengio Y. Deep learning. Cambridge: MIT press; 2016 Nov 18.
5. Chollet, F. Keras. 2015 (Version 1.2.2)[Computer software]. url: https://github.com/fchollet/keras
6. Abadi M, Barham P, Chen J, Chen Z, Davis A, Dean J, Devin M, Ghemawat S, Irving G, Isard M, Kudlur M. Tensorflow: a system for large-scale machine learning. InOSDI 2016 Nov 2 (Vol. 16, pp. 265-283).
7. Ahn WY, Kishida KT, Gu X, Lohrenz T, Harvey A, Alford JR, Smith KB, Yaffe G, Hibbing JR, Dayan P, Montague PR. Nonpolitical images evoke neural predictors of political ideology. Current Biology. 2014 Nov 17;24(22):2693-9.
8. JASP Team. JASP. 2016 (Version 0.8.0.0)[Computer software].
9. Marsman M, Wagenmakers EJ. Bayesian benefits with JASP. European Journal of Developmental Psychology. 2017 Sep 3;14(5):545-55.
10. Rouder JN, Speckman PL, Sun D, Morey RD, Iverson G. Bayesian t tests for accepting and rejecting the null hypothesis. Psychonomic bulletin & review. 2009 Apr 1;16(2):225-37.
11. Wagenmakers EJ, Morey RD, Lee MD. Bayesian benefits for the pragmatic researcher. Current Directions in Psychological Science. 2016 Jun;25(3):169-76.
